# *catena*-Poly[[[aqua­(pyrazine-2-carboxamide-κ^2^ *N* ^1^,*O*)zinc]-μ-pyrazine-2-carboxamide-κ^3^ *N* ^1^,*O*:*N* ^4^] dinitrate]

**DOI:** 10.1107/S1600536812016017

**Published:** 2012-04-18

**Authors:** Sadif A. Shirvan, Sara Haydari Dezfuli

**Affiliations:** a Department of Chemistry, Omidieh Branch, Islamic Azad University, Omidieh, Iran

## Abstract

In the crystal of the title compound, {[Zn(C_5_H_5_N_3_O)_2_(H_2_O)](NO_3_)_2_}_*n*_, the Zn^II^ cation is *N*,*O*-chelated by two pyrazine-2-carboxamide (PCA) ligands and is further coordinated by one water mol­ecule and by one pyrazine-N atom from an adjacent PCA ligand in a distorted ZnN_3_O_3_ octa­hedral geometry. One of the two independent PCA ligands bridges two Zn^II^ cations, forming a zigzag polymeric chain running along the *c* axis. In the crystal, the NO_3_^−^ anions link to the chain *via* O—H⋯O and N—H⋯O hydrogen bonding. Weak inter­molecular C—H⋯O inter­actions also occur.

## Related literature

For related structures, see: Shirvan & Haydari Dezfuli (2012 ▶); Abu-Youssef *et al.* (2006 ▶); Azhdari Tehrani *et al.* (2010 ▶); Goher & Mautner (2000 ▶); Kristiansson (2002 ▶); Mir Mohammad Sadegh *et al.* (2010 ▶); Munakata *et al.* (1997 ▶); Pacigova *et al.* (2008 ▶). 
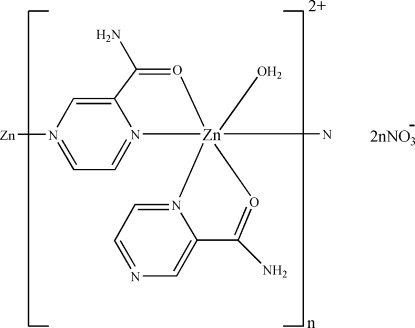


## Experimental

### 

#### Crystal data

[Zn(C_5_H_5_N_3_O)_2_(H_2_O)](NO_3_)_2_
*M* *_r_* = 453.67
Monoclinic, 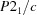

*a* = 10.4889 (11) Å
*b* = 15.7477 (16) Å
*c* = 9.9332 (10) Å
β = 97.664 (8)°
*V* = 1626.1 (3) Å^3^
*Z* = 4
Mo *K*α radiation
μ = 1.58 mm^−1^
*T* = 298 K
0.23 × 0.12 × 0.10 mm

#### Data collection

Bruker APEXII CCD area detector diffractometer
Absorption correction: multi-scan (*SADABS*; Bruker, 2001 ▶) *T*_min_ = 0.070, *T*_max_ = 0.240
9288 measured reflections
3192 independent reflections
2088 reflections with *I* > 2σ(*I*)
*R*_int_ = 0.123

#### Refinement

*R*[*F* ^2^ > 2σ(*F* ^2^)] = 0.076
*wR*(*F* ^2^) = 0.103
*S* = 1.06
3192 reflections
261 parameters
H atoms treated by a mixture of independent and constrained refinement
Δρ_max_ = 0.46 e Å^−3^
Δρ_min_ = −0.52 e Å^−3^

### 

Data collection: *APEX2* (Bruker, 2007 ▶); cell refinement: *SAINT* (Bruker, 2007 ▶); data reduction: *SAINT*; program(s) used to solve structure: *SHELXTL* (Sheldrick, 2008 ▶); program(s) used to refine structure: *SHELXTL*; molecular graphics: *SHELXTL*; software used to prepare material for publication: *SHELXTL*.

## Supplementary Material

Crystal structure: contains datablock(s) I, global. DOI: 10.1107/S1600536812016017/xu5508sup1.cif

Structure factors: contains datablock(s) I. DOI: 10.1107/S1600536812016017/xu5508Isup2.hkl

Additional supplementary materials:  crystallographic information; 3D view; checkCIF report

## Figures and Tables

**Table 1:** Selected bond lengths (Å)

Zn1—O1	2.064 (3)
Zn1—O2	2.073 (3)
Zn1—O3	2.042 (6)
Zn1—N1	2.180 (5)
Zn1—N4	2.193 (5)
Zn1—N5^i^	2.179 (5)

Symmetry code: (i) 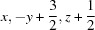.

**Table 2:** Hydrogen-bond geometry (Å, °)

*D*—H⋯*A*	*D*—H	H⋯*A*	*D*⋯*A*	*D*—H⋯*A*
O3—H3*B*⋯O8^ii^	0.76 (7)	2.05 (7)	2.810 (8)	177 (7)
O3—H3*C*⋯O4	0.75 (6)	2.06 (7)	2.781 (8)	162 (9)
N3—H3*D*⋯O3^ii^	0.86	2.50	3.193 (8)	138
N3—H3*D*⋯O5^ii^	0.86	2.42	3.163 (8)	144
N3—H3*E*⋯O7	0.86	2.08	2.937 (8)	172
N6—H6*B*⋯O4^iii^	0.86	2.07	2.913 (7)	166
N6—H6*C*⋯O5^iv^	0.86	2.41	3.231 (8)	161
C1—H1⋯O8^v^	0.93	2.39	3.292 (8)	162
C3—H3⋯O7	0.93	2.31	3.227 (8)	169
C6—H6⋯O6^vi^	0.93	2.60	3.295 (8)	132
C7—H7⋯O5^vi^	0.93	2.49	3.362 (8)	156
C8—H8⋯O4^iii^	0.93	2.39	3.298 (7)	167

Symmetry codes: (ii) 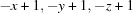; (iii) 
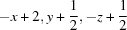
; (iv) 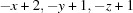; (v) 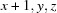; (vi) 
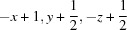
.

**Fig. 1.**
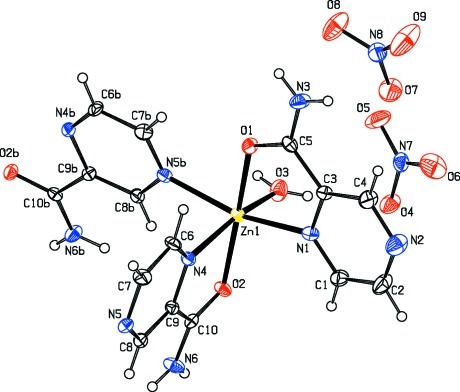
The molecular structure of the title molecule, with the atom-numbering scheme. Displacement ellipsoids are drawn at the 30% probability level. [Symmetry codes: (b) x,3/2 - y,1/2 + z].

**Fig. 2.**
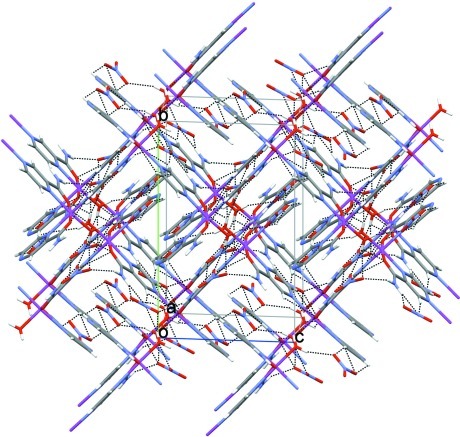
Unit-cell packing diagram for title molecule. Hydrogen bonds are shown as dashed lines.

## supplementary crystallographic information

### Comment

In a recent paper, we reported the synthesis and crystal structure of [ZnBr_2_(pzc)_2_] (Shirvan & Haydari Dezfuli, 2012), where pzc is the pyrazine-2-carboxamide. Pyrazine-2-carboxamide is a good ligand, and a few complexes with pzc have been prepared, such as that of mercury (Azhdari Tehrani *et al.*, 2010; Mir Mohammad Sadegh *et al.*, 2010) and vanadium (Pacigova *et al.*, 2008), manganese (Abu-Youssef *et al.*, 2006) and copper (Kristiansson, 2002; Munakata *et al.*, 1997; Goher & Mautner, 2000). Here, we report the synthesis and structure of the title compound.

The asymmetric unit of the title compound, (Fig. 1), contains one Zn^II^ cation, two pyrazine-2-carboxamide ligands, one water molecule and two NO_3_^-^ counter-ions. The Zn^II^ atom is six-coordinated in a distorted octahedral configuration by two N and two O atoms from two pyrazine-2-carboxamide ligands and one O atom from one water molecule. The sixth coordination site is occupied by N atom from one bridging pyrazine-2-carboxamide ligand. The Zn—O and Zn—N bond lengths and angles are collected in Table 1.

In the crystal structure, intra and intermolecular O—H···O, N—H···O and C—H···O hydrogen bonds (Table 2, Fig. 2) may stabilize the structure.

### Experimental

A solution of pyrazine-2-carboxamide (0.25 g, 2.0 mmol) in methanol (10 ml) was added to a solution of Zn(NO_3_)_2_.4H_2_O (0.26 g, 1.0 mmol) in methanol (10 ml) and the resulting colorless solution was stirred for 15 min at room temperature. This solution was left to evaporate slowly at room temperature. After one week, colorless block crystals of the title compound were isolated (yield 0.36 g, 79.3%).

### Refinement

Water H atoms were located in a difference Fourier map and refined isotropically. Other H atoms were positioned geometrically with C—H = 0.93 and N—H = 0.86 Å, and constrained to ride on their parent atoms with *U*_iso_(H) = 1.2*U*_eq_(N,C).

### Crystal data

**Table:**

[Zn(C_5_H_5_N_3_O)_2_(H_2_O)](NO_3_)_2_	*F*(000) = 920
*M**_r_* = 453.67	*D*_x_ = 1.853 Mg m^−^^3^
Monoclinic, *P*2_1_/*c*	Mo *K*α radiation, λ = 0.71073 Å
Hall symbol: -P 2ybc	Cell parameters from 9288 reflections
*a* = 10.4889 (11) Å	θ = 2.4–26.0°
*b* = 15.7477 (16) Å	µ = 1.58 mm^−^^1^
*c* = 9.9332 (10) Å	*T* = 298 K
β = 97.664 (8)°	Block, colorless
*V* = 1626.1 (3) Å^3^	0.23 × 0.12 × 0.10 mm
*Z* = 4	

### Data collection

**Table:**

Bruker APEXII CCD area detector diffractometer	3192 independent reflections
Radiation source: fine-focus sealed tube	2088 reflections with *I* > 2σ(*I*)
Graphite monochromator	*R*_int_ = 0.123
ω scans	θ_max_ = 26.0°, θ_min_ = 2.4°
Absorption correction: multi-scan (*SADABS*; Bruker, 2001)	*h* = −12→12
*T*_min_ = 0.070, *T*_max_ = 0.240	*k* = −19→19
9288 measured reflections	*l* = −12→11

### Refinement

**Table:**

Refinement on *F*^2^	Primary atom site location: structure-invariant direct methods
Least-squares matrix: full	Secondary atom site location: difference Fourier map
*R*[*F*^2^ > 2σ(*F*^2^)] = 0.076	Hydrogen site location: inferred from neighbouring sites
*wR*(*F*^2^) = 0.103	H atoms treated by a mixture of independent and constrained refinement
*S* = 1.06	*w* = 1/[σ^2^(*F*_o_^2^) + (0.0237*P*)^2^] where *P* = (*F*_o_^2^ + 2*F*_c_^2^)/3
3192 reflections	(Δ/σ)_max_ = 0.006
261 parameters	Δρ_max_ = 0.46 e Å^−^^3^
0 restraints	Δρ_min_ = −0.52 e Å^−^^3^

### Special details

**Table:**

Geometry. All e.s.d.'s (except the e.s.d. in the dihedral angle between two l.s. planes) are estimated using the full covariance matrix. The cell e.s.d.'s are taken into account individually in the estimation of e.s.d.'s in distances, angles and torsion angles; correlations between e.s.d.'s in cell parameters are only used when they are defined by crystal symmetry. An approximate (isotropic) treatment of cell e.s.d.'s is used for estimating e.s.d.'s involving l.s. planes.
Refinement. Refinement of *F*^2^ against ALL reflections. The weighted *R*-factor *wR* and goodness of fit *S* are based on *F*^2^, conventional *R*-factors *R* are based on *F*, with *F* set to zero for negative *F*^2^. The threshold expression of *F*^2^ > σ(*F*^2^) is used only for calculating *R*-factors(gt) *etc*. and is not relevant to the choice of reflections for refinement. *R*-factors based on *F*^2^ are statistically about twice as large as those based on *F*, and *R*- factors based on ALL data will be even larger.

### Fractional atomic coordinates and isotropic or equivalent isotropic displacement parameters (Å^2^)

**Table:**

	*x*	*y*	*z*	*U*_iso_*/*U*_eq_	
C1	0.7871 (6)	0.4587 (4)	0.1320 (7)	0.0329 (15)	
H1	0.8748	0.4701	0.1417	0.039*	
C2	0.7334 (6)	0.4048 (5)	0.0291 (7)	0.0445 (17)	
H2	0.7859	0.3819	−0.0300	0.053*	
C3	0.5393 (6)	0.4184 (4)	0.1012 (7)	0.0387 (16)	
H3	0.4526	0.4043	0.0946	0.046*	
C4	0.5906 (5)	0.4738 (3)	0.2038 (6)	0.0210 (12)	
C5	0.5169 (5)	0.5161 (3)	0.3017 (6)	0.0237 (13)	
C6	0.6418 (5)	0.7234 (4)	0.1580 (7)	0.0248 (14)	
H6	0.5624	0.7048	0.1782	0.030*	
C7	0.6463 (5)	0.7868 (4)	0.0610 (7)	0.0292 (15)	
H7	0.5699	0.8084	0.0154	0.035*	
C8	0.8651 (5)	0.7831 (3)	0.0990 (6)	0.0240 (14)	
H8	0.9445	0.8033	0.0814	0.029*	
C9	0.8615 (5)	0.7189 (3)	0.1932 (6)	0.0189 (12)	
C10	0.9758 (5)	0.6759 (3)	0.2712 (6)	0.0207 (13)	
N1	0.7158 (4)	0.4940 (3)	0.2159 (5)	0.0246 (12)	
N2	0.6089 (5)	0.3848 (4)	0.0128 (6)	0.0483 (16)	
N3	0.3939 (5)	0.5002 (3)	0.3014 (6)	0.0366 (14)	
H3D	0.3515	0.5264	0.3571	0.044*	
H3E	0.3558	0.4637	0.2454	0.044*	
N4	0.7479 (4)	0.6893 (3)	0.2219 (5)	0.0228 (12)	
N5	0.7584 (4)	0.8170 (3)	0.0325 (5)	0.0225 (11)	
N6	1.0915 (4)	0.7048 (3)	0.2613 (5)	0.0317 (13)	
H6C	1.1576	0.6814	0.3071	0.038*	
H6B	1.1012	0.7472	0.2090	0.038*	
N7	0.7146 (5)	0.3192 (3)	0.4190 (7)	0.0350 (13)	
N8	0.1273 (5)	0.4024 (4)	0.1241 (6)	0.0361 (13)	
O1	0.5749 (3)	0.5684 (2)	0.3812 (4)	0.0300 (11)	
O2	0.9561 (3)	0.6152 (2)	0.3448 (4)	0.0252 (9)	
O3	0.8371 (5)	0.5012 (4)	0.5193 (6)	0.0397 (13)	
H3C	0.850 (7)	0.456 (4)	0.503 (8)	0.04 (3)*	
H3B	0.853 (6)	0.510 (4)	0.595 (7)	0.03 (2)*	
O4	0.8335 (4)	0.3362 (3)	0.4190 (5)	0.0437 (12)	
O5	0.6640 (5)	0.3426 (4)	0.5181 (7)	0.080 (2)	
O6	0.6571 (5)	0.2815 (4)	0.3235 (6)	0.0633 (16)	
O7	0.2393 (5)	0.3816 (3)	0.1188 (7)	0.0622 (15)	
O8	0.1027 (5)	0.4586 (4)	0.2039 (6)	0.0669 (17)	
O9	0.0400 (5)	0.3687 (4)	0.0522 (8)	0.098 (3)	
Zn1	0.76855 (6)	0.58798 (4)	0.37477 (7)	0.02037 (17)	

### Atomic displacement parameters (Å^2^)

**Table:**

	*U*^11^	*U*^22^	*U*^33^	*U*^12^	*U*^13^	*U*^23^
C1	0.021 (3)	0.037 (3)	0.042 (4)	0.001 (3)	0.007 (3)	−0.005 (3)
C2	0.044 (4)	0.049 (4)	0.041 (4)	0.003 (4)	0.008 (3)	−0.020 (4)
C3	0.030 (3)	0.048 (4)	0.038 (4)	−0.012 (3)	0.004 (3)	−0.020 (4)
C4	0.020 (3)	0.028 (3)	0.015 (3)	−0.003 (2)	0.000 (2)	−0.001 (3)
C5	0.023 (3)	0.023 (3)	0.025 (4)	−0.003 (2)	−0.001 (3)	0.005 (3)
C6	0.018 (3)	0.025 (3)	0.033 (4)	−0.004 (2)	0.007 (3)	0.006 (3)
C7	0.013 (3)	0.035 (4)	0.039 (4)	0.001 (2)	0.001 (3)	0.003 (3)
C8	0.021 (3)	0.019 (3)	0.033 (4)	0.001 (2)	0.007 (3)	−0.001 (3)
C9	0.017 (3)	0.020 (3)	0.018 (3)	−0.002 (2)	0.001 (2)	−0.001 (2)
C10	0.022 (3)	0.021 (3)	0.020 (3)	0.003 (2)	0.004 (2)	−0.004 (3)
N1	0.021 (2)	0.030 (3)	0.023 (3)	−0.001 (2)	0.003 (2)	0.003 (2)
N2	0.038 (3)	0.060 (4)	0.046 (4)	−0.004 (3)	0.003 (3)	−0.021 (3)
N3	0.023 (3)	0.052 (3)	0.035 (4)	−0.004 (2)	0.009 (2)	−0.011 (3)
N4	0.020 (2)	0.020 (3)	0.029 (3)	0.000 (2)	0.006 (2)	0.003 (2)
N5	0.022 (2)	0.024 (2)	0.021 (3)	0.005 (2)	0.004 (2)	−0.002 (2)
N6	0.015 (2)	0.039 (3)	0.041 (4)	0.001 (2)	0.003 (2)	0.017 (3)
N7	0.027 (3)	0.025 (3)	0.053 (4)	0.002 (2)	0.004 (3)	−0.004 (3)
N8	0.030 (3)	0.035 (3)	0.042 (4)	0.003 (3)	0.002 (3)	0.001 (3)
O1	0.022 (2)	0.034 (3)	0.035 (3)	−0.0099 (18)	0.0051 (19)	−0.017 (2)
O2	0.020 (2)	0.025 (2)	0.031 (3)	0.0002 (16)	0.0029 (18)	0.0098 (18)
O3	0.053 (3)	0.034 (3)	0.030 (4)	0.009 (3)	0.000 (3)	0.005 (3)
O4	0.024 (2)	0.052 (3)	0.056 (4)	0.001 (2)	0.007 (2)	−0.017 (3)
O5	0.063 (4)	0.087 (4)	0.103 (5)	−0.015 (3)	0.057 (4)	−0.041 (4)
O6	0.045 (3)	0.077 (4)	0.063 (4)	−0.022 (3)	−0.009 (3)	−0.006 (3)
O7	0.028 (3)	0.065 (3)	0.094 (4)	−0.006 (3)	0.010 (3)	−0.022 (3)
O8	0.052 (3)	0.088 (4)	0.056 (4)	0.018 (3)	−0.009 (3)	−0.039 (3)
O9	0.026 (3)	0.135 (6)	0.127 (6)	0.003 (3)	−0.007 (3)	−0.092 (5)
Zn1	0.0182 (3)	0.0208 (3)	0.0216 (3)	−0.0026 (3)	0.0009 (2)	−0.0004 (4)

### Geometric parameters (Å, º)

**Table:**

Zn1—O1	2.064 (3)	N4—C9	1.345 (7)
Zn1—O2	2.073 (3)	N4—C6	1.320 (7)
Zn1—O3	2.042 (6)	N5—C8	1.333 (7)
Zn1—N1	2.180 (5)	N5—C7	1.333 (7)
Zn1—N4	2.193 (5)	N6—C10	1.312 (7)
Zn1—N5^i^	2.179 (5)	N3—H3E	0.8600
O1—C5	1.243 (6)	N3—H3D	0.8600
O2—C10	1.237 (6)	N6—H6B	0.8600
O3—H3B	0.76 (7)	N6—H6C	0.8600
O3—H3C	0.75 (6)	C1—C2	1.389 (10)
O4—N7	1.276 (7)	C3—C4	1.394 (9)
O5—N7	1.235 (9)	C4—C5	1.479 (8)
O6—N7	1.210 (9)	C6—C7	1.393 (9)
O7—N8	1.227 (8)	C8—C9	1.382 (7)
O8—N8	1.238 (9)	C9—C10	1.500 (8)
O9—N8	1.207 (9)	C1—H1	0.9300
N1—C4	1.341 (7)	C2—H2	0.9300
N1—C1	1.315 (8)	C3—H3	0.9300
N2—C2	1.332 (8)	C6—H6	0.9300
N2—C3	1.325 (9)	C7—H7	0.9300
N3—C5	1.314 (7)	C8—H8	0.9300
			
Zn1···H3D^ii^	3.5800	N4···C10	2.385 (7)
O1···O3	3.092 (6)	N4···N5^i^	3.074 (7)
O1···N1	2.627 (6)	N5···N4^viii^	3.074 (7)
O1···N4	3.192 (6)	N5···O3^viii^	2.987 (8)
O1···C4	2.330 (7)	N5···O1^viii^	2.907 (6)
O1···O5^ii^	3.148 (7)	N5···N4	2.766 (7)
O1···N5^i^	2.907 (6)	N5···O2^viii^	3.154 (6)
O1···C7^i^	2.932 (7)	N6···O6^ix^	3.117 (7)
O2···C9	2.350 (6)	N6···O4^ix^	2.913 (7)
O2···O3	2.892 (7)	N6···O4^iii^	3.233 (7)
O2···N4	2.629 (6)	N6···O5^iii^	3.231 (8)
O2···C8^i^	3.240 (7)	N7···C4	3.383 (8)
O2···O3^iii^	3.018 (7)	N7···O3	3.243 (8)
O2···O4^iii^	3.095 (6)	N8···C8^v^	3.323 (8)
O2···N5^i^	3.154 (6)	N8···C9^v^	3.406 (8)
O3···N1	3.113 (8)	N2···H6^vii^	2.8100
O3···O5	3.087 (8)	N3···H3B^ii^	2.92 (6)
O3···N5^i^	2.987 (8)	N3···H3	2.6900
O3···O2^iii^	3.018 (7)	N5···H3B^viii^	2.94 (6)
O3···O1	3.092 (6)	N6···H8	2.6900
O3···O2	2.892 (7)	N7···H6C^iii^	2.8700
O3···O4	2.781 (8)	N7···H3C	2.65 (7)
O3···O8^ii^	2.810 (8)	N7···H6B^iv^	2.7000
O3···N7	3.243 (8)	N8···H1^vi^	2.8800
O3···N3^ii^	3.193 (8)	N8···H3E	2.7100
O4···O3	2.781 (8)	C1···O9^x^	3.199 (9)
O4···C8^iv^	3.298 (7)	C1···O8^x^	3.292 (8)
O4···N6^iii^	3.233 (7)	C2···O9^x^	3.244 (8)
O4···O2^iii^	3.095 (6)	C3···O6	3.213 (9)
O4···N6^iv^	2.913 (7)	C3···O7	3.227 (8)
O5···C7^v^	3.362 (8)	C3···C4^vii^	3.580 (9)
O5···N6^iii^	3.231 (8)	C3···C3^vii^	3.299 (9)
O5···N3^ii^	3.163 (8)	C4···C3^vii^	3.580 (9)
O5···O1^ii^	3.148 (7)	C4···O6	3.294 (8)
O5···O3	3.087 (8)	C4···N7	3.383 (8)
O6···N6^iv^	3.117 (7)	C6···N2^vii^	3.392 (8)
O6···C4	3.294 (8)	C6···O6^xi^	3.295 (8)
O6···C3	3.213 (9)	C7···O5^xi^	3.362 (8)
O6···C6^v^	3.295 (8)	C8···N8^xi^	3.323 (8)
O7···C3	3.227 (8)	C8···O9^vii^	3.059 (8)
O7···N3	2.937 (8)	C8···O8^xi^	3.378 (8)
O8···C8^v^	3.378 (8)	C8···O4^ix^	3.298 (7)
O8···C1^vi^	3.292 (8)	C9···N8^xi^	3.406 (8)
O8···N3	3.148 (8)	C9···O9^vii^	3.096 (9)
O8···O3^ii^	2.810 (8)	C10···O9^vii^	3.271 (10)
O9···C9^vii^	3.096 (9)	C3···H3E	2.6500
O9···C2^vi^	3.244 (8)	C8···H6B	2.6300
O9···C8^vii^	3.059 (8)	H1···O8^x^	2.3900
O9···C1^vi^	3.199 (9)	H1···O9^x^	2.6000
O9···C10^vii^	3.271 (10)	H1···N8^x^	2.8800
O1···H7^i^	2.3600	H2···O9^x^	2.6900
O2···H3C^iii^	2.62 (7)	H3···N3	2.6900
O2···H3B^iii^	2.82 (6)	H3···H3E	2.1300
O2···H8^i^	2.6900	H3···O7	2.3100
O3···H3D^ii^	2.5000	H3B···O2^iii^	2.82 (6)
O4···H3C	2.06 (7)	H3B···H3D^ii^	2.3300
O4···H6C^iii^	2.7200	H3B···O8^ii^	2.05 (7)
O4···H6B^iv^	2.0700	H3B···N3^ii^	2.92 (6)
O4···H8^iv^	2.3900	H3C···O5	2.66 (7)
O5···H3C	2.66 (7)	H3C···O4	2.06 (7)
O5···H6C^iii^	2.4100	H3C···N7	2.65 (7)
O5···H7^v^	2.4900	H3C···O2^iii^	2.62 (7)
O5···H3D^ii^	2.4200	H3D···Zn1^ii^	3.5800
O6···H6^v^	2.6000	H3D···H3B^ii^	2.3300
O6···H6B^iv^	2.6500	H3D···O3^ii^	2.5000
O7···H3	2.3100	H3D···O5^ii^	2.4200
O7···H3E	2.0800	H3E···O7	2.0800
O8···H3E	2.6300	H3E···O8	2.6300
O8···H1^vi^	2.3900	H3E···N8	2.7100
O8···H3B^ii^	2.05 (7)	H3E···C3	2.6500
O9···H2^vi^	2.6900	H3E···H3	2.1300
O9···H1^vi^	2.6000	H6···N2^vii^	2.8100
N1···C5	2.382 (7)	H6···O6^xi^	2.6000
N1···O1	2.627 (6)	H6B···O6^ix^	2.6500
N1···N2	2.772 (8)	H6B···O4^ix^	2.0700
N1···N4	3.094 (7)	H6B···N7^ix^	2.7000
N1···O3	3.113 (8)	H6B···H8	2.1300
N2···N1	2.772 (8)	H6B···C8	2.6300
N2···C6^vii^	3.392 (8)	H6C···O5^iii^	2.4100
N3···O5^ii^	3.163 (8)	H6C···O4^iii^	2.7200
N3···O7	2.937 (8)	H6C···N7^iii^	2.8700
N3···O3^ii^	3.193 (8)	H7···O5^xi^	2.4900
N3···O8	3.148 (8)	H7···O1^viii^	2.3600
N4···N1	3.094 (7)	H8···O4^ix^	2.3900
N4···O1	3.192 (6)	H8···O2^viii^	2.6900
N4···O2	2.629 (6)	H8···N6	2.6900
N4···N5	2.766 (7)	H8···H6B	2.1300
			
O1—Zn1—O2	172.71 (15)	O4—N7—O5	117.5 (6)
O1—Zn1—O3	97.70 (18)	O4—N7—O6	119.2 (6)
O1—Zn1—N1	76.42 (15)	O5—N7—O6	123.3 (6)
O1—Zn1—N4	97.09 (15)	O8—N8—O9	119.1 (6)
O1—Zn1—N5^i^	86.43 (15)	O7—N8—O8	120.0 (6)
O2—Zn1—O3	89.28 (18)	O7—N8—O9	120.8 (6)
O2—Zn1—N1	100.98 (15)	N1—C1—C2	121.0 (6)
O2—Zn1—N4	76.01 (15)	N2—C2—C1	121.9 (6)
O2—Zn1—N5^i^	95.71 (15)	N2—C3—C4	122.8 (6)
O3—Zn1—N1	94.9 (2)	C3—C4—C5	125.3 (5)
O3—Zn1—N4	165.13 (19)	N1—C4—C5	115.2 (5)
O3—Zn1—N5^i^	90.0 (2)	N1—C4—C3	119.5 (5)
N1—Zn1—N4	90.03 (18)	O1—C5—C4	117.5 (5)
N1—Zn1—N5^i^	162.62 (16)	O1—C5—N3	121.7 (5)
N4—Zn1—N5^i^	89.34 (18)	N3—C5—C4	120.8 (5)
Zn1—O1—C5	118.7 (3)	N4—C6—C7	121.4 (5)
Zn1—O2—C10	118.8 (3)	N5—C7—C6	121.0 (5)
H3B—O3—H3C	112 (8)	N5—C8—C9	122.1 (5)
Zn1—O3—H3C	123 (6)	C8—C9—C10	126.0 (5)
Zn1—O3—H3B	126 (5)	N4—C9—C10	113.8 (5)
C1—N1—C4	118.4 (5)	N4—C9—C8	120.2 (5)
Zn1—N1—C4	112.1 (4)	O2—C10—C9	118.0 (5)
Zn1—N1—C1	129.4 (4)	N6—C10—C9	119.2 (5)
C2—N2—C3	116.3 (6)	O2—C10—N6	122.8 (5)
Zn1—N4—C6	128.9 (4)	C2—C1—H1	120.00
Zn1—N4—C9	113.0 (3)	N1—C1—H1	119.00
C6—N4—C9	118.1 (5)	N2—C2—H2	119.00
Zn1^viii^—N5—C8	120.9 (4)	C1—C2—H2	119.00
C7—N5—C8	117.3 (5)	N2—C3—H3	119.00
Zn1^viii^—N5—C7	121.8 (4)	C4—C3—H3	119.00
C5—N3—H3E	120.00	C7—C6—H6	119.00
H3D—N3—H3E	120.00	N4—C6—H6	119.00
C5—N3—H3D	120.00	N5—C7—H7	120.00
C10—N6—H6B	120.00	C6—C7—H7	119.00
C10—N6—H6C	120.00	C9—C8—H8	119.00
H6B—N6—H6C	120.00	N5—C8—H8	119.00
			
O3—Zn1—O1—C5	−92.4 (4)	Zn1—O2—C10—N6	170.5 (4)
N1—Zn1—O1—C5	0.9 (4)	Zn1—O2—C10—C9	−8.1 (6)
N4—Zn1—O1—C5	89.2 (4)	Zn1—N1—C4—C5	−2.1 (6)
N5^i^—Zn1—O1—C5	178.1 (4)	C1—N1—C4—C3	−1.8 (8)
O3—Zn1—O2—C10	−171.7 (4)	C4—N1—C1—C2	3.0 (9)
N1—Zn1—O2—C10	93.4 (4)	Zn1—N1—C4—C3	176.1 (4)
N4—Zn1—O2—C10	6.2 (4)	Zn1—N1—C1—C2	−174.5 (5)
N5^i^—Zn1—O2—C10	−81.7 (4)	C1—N1—C4—C5	−180.0 (5)
O1—Zn1—N1—C1	178.4 (6)	C2—N2—C3—C4	1.9 (10)
O2—Zn1—N1—C1	5.4 (6)	C3—N2—C2—C1	−0.8 (10)
O3—Zn1—N1—C1	−84.9 (5)	Zn1—N4—C9—C8	−179.9 (4)
N4—Zn1—N1—C1	81.1 (5)	Zn1—N4—C6—C7	−178.8 (5)
O1—Zn1—N1—C4	0.8 (4)	C6—N4—C9—C10	−180.0 (5)
O2—Zn1—N1—C4	−172.3 (4)	Zn1—N4—C9—C10	0.3 (6)
O3—Zn1—N1—C4	97.5 (4)	C6—N4—C9—C8	−0.1 (8)
N4—Zn1—N1—C4	−96.5 (4)	C9—N4—C6—C7	1.6 (9)
O1—Zn1—N4—C6	−0.4 (5)	C8—N5—C7—C6	1.0 (9)
O2—Zn1—N4—C6	177.3 (6)	Zn1^viii^—N5—C7—C6	177.2 (5)
N1—Zn1—N4—C6	76.0 (5)	Zn1^viii^—N5—C8—C9	−175.8 (4)
N5^i^—Zn1—N4—C6	−86.7 (5)	C7—N5—C8—C9	0.4 (8)
O1—Zn1—N4—C9	179.3 (4)	N1—C1—C2—N2	−1.7 (11)
O2—Zn1—N4—C9	−3.0 (4)	N2—C3—C4—C5	177.3 (6)
N1—Zn1—N4—C9	−104.3 (4)	N2—C3—C4—N1	−0.7 (9)
N5^i^—Zn1—N4—C9	93.0 (4)	N1—C4—C5—N3	−178.8 (5)
O1^viii^—Zn1^viii^—N5—C7	14.9 (5)	N1—C4—C5—O1	3.0 (7)
O2^viii^—Zn1^viii^—N5—C7	−158.1 (5)	C3—C4—C5—N3	3.2 (9)
O3^viii^—Zn1^viii^—N5—C7	112.6 (5)	C3—C4—C5—O1	−175.1 (5)
N4^viii^—Zn1^viii^—N5—C7	−82.2 (5)	N4—C6—C7—N5	−2.1 (10)
O1^viii^—Zn1^viii^—N5—C8	−169.1 (4)	N5—C8—C9—N4	−0.9 (8)
O2^viii^—Zn1^viii^—N5—C8	17.9 (4)	N5—C8—C9—C10	178.9 (5)
O3^viii^—Zn1^viii^—N5—C8	−71.4 (4)	N4—C9—C10—O2	5.0 (7)
N4^viii^—Zn1^viii^—N5—C8	93.8 (4)	C8—C9—C10—N6	6.5 (8)
Zn1—O1—C5—N3	179.4 (4)	N4—C9—C10—N6	−173.6 (5)
Zn1—O1—C5—C4	−2.3 (6)	C8—C9—C10—O2	−174.9 (5)

Symmetry codes: (i) *x*, −*y*+3/2, *z*+1/2; (ii) −*x*+1, −*y*+1, −*z*+1; (iii) −*x*+2, −*y*+1, −*z*+1; (iv) −*x*+2, *y*−1/2, −*z*+1/2; (v) −*x*+1, *y*−1/2, −*z*+1/2; (vi) *x*−1, *y*, *z*; (vii) −*x*+1, −*y*+1, −*z*; (viii) *x*, −*y*+3/2, *z*−1/2; (ix) −*x*+2, *y*+1/2, −*z*+1/2; (x) *x*+1, *y*, *z*; (xi) −*x*+1, *y*+1/2, −*z*+1/2.

### Hydrogen-bond geometry (Å, º)

**Table:**

*D*—H···*A*	*D*—H	H···*A*	*D*···*A*	*D*—H···*A*
O3—H3*B*···O8^ii^	0.76 (7)	2.05 (7)	2.810 (8)	177 (7)
O3—H3*C*···O4	0.75 (6)	2.06 (7)	2.781 (8)	162 (9)
N3—H3*D*···O3^ii^	0.86	2.50	3.193 (8)	138
N3—H3*D*···O5^ii^	0.86	2.42	3.163 (8)	144
N3—H3*E*···O7	0.86	2.08	2.937 (8)	172
N6—H6*B*···O4^ix^	0.86	2.07	2.913 (7)	166
N6—H6*C*···O5^iii^	0.86	2.41	3.231 (8)	161
C1—H1···O8^x^	0.93	2.39	3.292 (8)	162
C3—H3···O7	0.93	2.31	3.227 (8)	169
C6—H6···O6^xi^	0.93	2.60	3.295 (8)	132
C7—H7···O5^xi^	0.93	2.49	3.362 (8)	156
C8—H8···O4^ix^	0.93	2.39	3.298 (7)	167

Symmetry codes: (ii) −*x*+1, −*y*+1, −*z*+1; (iii) −*x*+2, −*y*+1, −*z*+1; (ix) −*x*+2, *y*+1/2, −*z*+1/2; (x) *x*+1, *y*, *z*; (xi) −*x*+1, *y*+1/2, −*z*+1/2.
